# Microscopic inflammation in ileocecal specimen does not correspond to a higher anastomotic leakage rate after ileocecal resection in Crohn’s disease

**DOI:** 10.1371/journal.pone.0247796

**Published:** 2021-03-04

**Authors:** Christian Schineis, Andrea Ullrich, Kai S. Lehmann, Christoph Holmer, Johannes C. Lauscher, Benjamin Weixler, Martin E. Kreis, Claudia Seifarth

**Affiliations:** 1 Department of Surgery, Charité – Universitätsmedizin Berlin, Corporate Member of Freie Universität Berlin, Humboldt-Universität zu Berlin, and Berlin Institute of Health, Berlin, Germany; 2 Department of Pathology, Charité – Universitätsmedizin Berlin, Corporate Member of Freie Universität Berlin, Humboldt-Universität zu Berlin, and Berlin Institute of Health, Berlin, Germany; 3 Department of Surgery, St. Joseph Hospital, Berlin, Germany; McMaster University, CANADA

## Abstract

**Background:**

Patients with Crohn’s disease suffer from a higher rate of anastomotic leakages after ileocecal resection than patients without Crohn’s disease. Our hypothesis was that microscopic inflammation at the resection margins of ileocecal resections in Crohn’s disease increases the rate of anastomotic leakages.

**Patients and methods:**

In a retrospective cohort study, 130 patients with Crohn’s disease that underwent ileocecal resection between 2015 and 2019, were analyzed. Anastomotic leakage was the primary outcome parameter. Inflammation at the resection margin was characterized as “inflammation at proximal resection margin”, “inflammation at distal resection margin” or “inflammation at both ends”.

**Results:**

46 patients (35.4%) showed microscopic inflammation at the resection margins. 17 patients (13.1%) developed anastomotic leakage. No difference in the rate of anastomotic leakages was found for proximally affected resection margins (no anastomotic leakage vs. anastomotic leakage: 20.3 vs. 35.3%, p = 0.17), distally affected resection margins (2.7 vs. 5.9%, p = 0.47) or inflammation at both ends (9.7 vs. 11.8%, p = 0.80). No effect on the anastomotic leakage rate was found for preoperative hemoglobin concentration (no anastomotic leakage vs. anastomotic leakage: 12.3 vs. 13.5 g/dl, p = 0.26), perioperative immunosuppressive medication (62.8 vs. 52.9%, p = 0.30), BMI (21.8 vs. 22.4 m^2^/kg, p = 0.82), emergency operation (21.2 vs. 11.8%, p = 0.29), laparoscopic vs. open procedure (p = 0.58), diverting ileostomy (31.9 vs. 57.1%, p = 0.35) or the level of surgical training (staff surgeon: 80.5 vs. 76.5%, p = 0.45).

**Conclusion:**

Microscopic inflammation at the resection margins after ileocecal resection in Crohn’s disease is common. Histologically inflamed resection margins do not appear to affect the rate of anastomotic leakages. Our data suggest that there is no need for extensive resections or frozen section to achieve microscopically inflammation-free resection margins.

## Introduction

The incidence of inflammatory bowel diseases (IBD) is continually rising in industrialized countries. As a result, more patients have to undergo surgical treatment secondary to fibrous stenosis, intestinal obstruction, fistulas, perforations or fulminant flare-ups that are refractory to medical treatment [1]. In the case of Crohn’s disease (CD), segmental resections in small intestine or colon often are carried out. As the terminal ileum is most frequently affected in patients with CD [2], ileocecal resection (ICR) is an established standard operation and the most frequent abdominal procedure in CD patients.

Recent publications show rates of anastomotic leakages after ICR of up to 17% in high risk CD patients [3–7]. Immunosuppression, reduced general health condition, reduced nutritional status, low albumin level and high doses of perioperative corticosteroid medication are known as risk factors for increased postoperative complications [8–10]. How to establish the ileocolic anastomosis in the safest possible manner is still a matter of debate and investigation [11–13]. Another possible cause of impaired anastomotic healing is inflammation at resection margins. Although recent studies have examined the influence of microscopic inflammation on the recurrence of CD at the resection margins and at the site of the anastomosis, few studies have examined the role of microscopic inflammation at the resection margins in relation to postoperative surgical complications [7, 14]. In a recently published article, authors demonstrated higher rates of anastomotic leakage when inflammation was present at the proximal resection margin [3].

In this study, we will analyze the effect of microscopic inflammation at the resection margins on postoperative anastomotic healing in patients with ileocecal resection in Crohn’s disease.

## Patients and methods

### Patients

In our department of Surgery, Charité—Universitätsmedizin Berlin, Campus Benjamin Franklin, a tertiary referral center for IBD, all patients who received an ICR for CD from January 1, 2015 to December 31, 2019, were included in this study. From March to August 2020, we accessed the retrospective data and fully evaluated them. Patients without anastomosis and patients with multiple resections in addition to ICR were excluded. The indication for surgery in all patients was based on the recommendation of the ECCO (European Crohn’s and Colitis Organization) guidelines for perforation, fistula, abscess formation, and stenosis in CD. In elective surgery, treatment with biologicals was ceased at least two weeks preoperatively in accordance with the recommendations of the ECCO guidelines. Steroids were reduced to the Cushing’s threshold level.

### Analysis

The study was designed as a retrospective cohort study. All data were collected from the hospital’s electronic health records (EHS) system. The primary outcome parameter was anastomotic leakage. It was defined as a local abscess at the anastomosis and/or outflow of intestinal contents into the abdominal cavity resulting in surgical revision or interventional drainage. Perioperative data such as age, diagnosis, gender, immunosuppressive medication, ASA (American Society of Anesthesiologists) score, body mass index (BMI) and the level of surgical training of the operating team were documented.

Inflammation at the resection margin was defined as transmural inflammation consisting of lymphocytes, plasma cells and macrophages and, depending on the activity of the inflammation, also ulcers and neutrophil granulocytes. A board-certified pathologist performed the evaluation of the resection margins.

### Interventions

All operations were performed in a standardized manner according to the operating procedures of our department. Every patient received a single shot perioperative antibiotic prophylaxis with a combination of 1.5 g Cefuroxim (M.P.I. Pharmaceutica, Hamburg, Germany) i.v. and 500 mg Metronidazole (Braun, Bethlehem, PA, USA) i.v. All resections were performed on the resection margins that were macroscopically free of inflammation.

Anastomoses were either performed as a side-to-side stapled anastomosis (Ethicon Proximate linear cutter 75, Johnson & Johnson Medical GmbH Germany; Stapler height 1.5 mm, 4 staple rows) or as side-to-side hand-sewn running suture anastomosis with PDS 4–0 (monofilament, absorbable, Polydiaxanon, Ethicon, Johnson & Johnson Medical GmbH, Norderstedt, Germany). The choice of the anastomotic technique was left to the operating surgeons´ discretion.

Patients were discharged as soon as normal diet and regular bowel movement were achieved, lab work was within normal range and mild pain was well controlled with oral analgesics.

Clinically relevant anastomotic leakages requiring reoperation or interventional treatment were recorded. Patients that were readmitted due to an anastomotic problem within 30 days after hospital discharge were included and taken into account.

### Statistical analysis

Since most variables showed skewed distributions, non-parametric tests were used for statistical comparison. Descriptive analyses included absolute and relative frequencies for categorical variables and median (minimum—maximum) for continuous variables. The Chi-Square-Test or Fisher’s exact test was used for univariate group comparisons of categorical variables. The Mann-Whitney U test was used for the comparison of continuous variables between groups. Statistical analyses were performed with SPSS Statistics Software 25.0 (IBM Armonk, NY, USA). P values of less than 0.05 (two-sided asymptotic significance) were defined as statistically significant.

### Ethics

The Ethics committee of the Charité—Universitätsmedizin Berlin approved the study protocol (EA4/197/18). Since this is a retrospective study, a written declaration of consent from the patient based on the legal basis of Section 25 of the State Hospital Law (LKG Berlin) is not required.

## Results

### Demographics

Between January 1, 2015 and December 31, 2019, 145 patients with CD who had undergone an ICR were analyzed. Fifteen patients were excluded due to a lack of information on inflammation in the histological examination or due to an anastomotic technique other than those described above. The remaining 130 patients were included in the evaluation.

The median age at operation was 36 years (13–78). 56.9% of the patients were female. Most patients (90.8%) were categorized ASA 1 and 2, BMI was 21.8 m^2^/kg (14.4–47) and preoperative hemoglobin concentration was 12.5 mg/dl (8–16.7). Surgery was performed with immunosuppression in 61.5% of the patients. About half of the operations (53.1%) were performed laparoscopically, the remainder were open (30%) or converted to open procedures (16.9%). A diverting ileostomy was formed in 30.8% of all cases. 80% of the operations were performed by a staff surgeon. The anastomotic leakage rate was 13.1%. No deaths occurred (Table 1).

**Table 1:** Demographics.

	n = 130
Age, years	36 (13–78)
BMI, m^2^/kg	21.8 (14.4–47)
Sex, female	74 (56.9)
ASA, 1–2	118 (90.8)
Missing	3
Hemoglobin preoperative, mg/dl	12.5 (8–16.7)
Immunosuppression	80 (61.5)
Surgical technique	
Laparoscopic	69 (53.1)
Open	39 (30)
Conversion	22 (16.9)
Diverting ileostomy	40 (30.8)
Operating surgeon	
Staff surgeon	104 (80)
Resident	26 (20)

### Effect of inflammation at the resection margins on anastomotic leakages after ileocecal resection

As shown in Table 2, no effect on the anastomotic leakage rate was found for microscopic inflammation at the proximal resection margin (no anastomotic leakage vs. anastomotic leakage: 20.3 vs. 35.3%, p = 0.17), inflammation at the distal resection margin (2.7 vs. 5.9%, p = 0.47) or inflammation at both ends (9.7 vs. 11.8%, p = 0.80). The remainder (n = 10) were free of inflammation. The analysis for any type of inflammation (proximal, distal or both resection margins) at the resection margin showed no significant difference (p = 0.11) for anastomotic leakages.

**Table 2:** Effect of inflammation at the resection margins on anastomotic leakages after ileocecal resection.

	No anastomotic leakage, n = 113	Anastomotic leakage, n = 17	p-value
Inflammation at proximal resection margin	23 (20.3)	6 (35.3)	0.17
Inflammation at distal resection margin	3 (2.7)	1 (5.9)	0.47
Inflammation at both ends	11 (9.7)	2 (11.8)	0.80
Any inflammation	37 (32.7)	9 (52.9)	0.11

### Perioperative risk factors for anastomotic leakages

Patient characteristics in the “anastomotic leakage” and “no anastomotic leakage” groups were well balanced concerning age (p = 0.12), BMI (p = 0.82), sex (p = 0.54) and ASA (p = 0.66). No significant influence of preoperative Hb concentration (p = 0.26), perioperative immunosuppressant medication (p = 0.30) or emergency operation (p = 0.29) on anastomotic leakages were found. Neither laparoscopic, open nor converted surgical approach (p = 0.58), diverting ileostomy (p = 0.35) nor the level of surgical training (p = 0.45) were related to the leakage rate (Table 3).

**Table 3:** Perioperative risk factors for the development of anastomotic leakages.

	No anastomotic leakage (n = 113)	Anastomotic leakage (n = 17)	p-value
Age, years	36 (13–78)	32 (14–61)	0.12
BMI, m^2^/kg	21.8 (14.4–47)	22.4 (16.2–29)	0.82
Sex, female	64 (56.6)	10 (58.8)	0.54
ASA, 1–2	110 (97.3)	16 (94.1)	0.66
Hemoglobin, mg/dl	12.3 (8–16.7)	13.5 (9.2–15)	0.26
Immunosuppression	71 (62.8)	9 (52.9)	0.30
Emergency procedure	24 (21.2)	2 (11.8)	0.29
Surgical technique			0.58
Laparoscopic	58 (51.3)	11 (64.7)	
Open	35 (31)	4 (23.5)	
Conversion	20 (17.7)	2 (11.8)	
Diverting ileostomy	36 (31.9)	4 (57.1)	0.35
Main surgeon			0.45
Staff surgeon	91 (80.5)	13 (76.5)	
Resident	22 (19.5)	4 (23.5)	

## Discussion

Patients with Crohn’s disease (CD) are known to have an increased risk of anastomotic complications [15–17]. The aim of our study was to investigate the influence of microscopic inflammation on anastomotic healing after ileocecal resections (ICR) in patients with CD.

We could show that microscopic inflammation at the resection margins was quite common with 35% in all patients. Our data also showed that there is no difference in the occurrence of anastomotic leakages whether microscopic inflammation is present or not. In addition, perioperative data such as age, gender, immunosuppressive medication, ASA score, BMI, preoperative hemoglobin level, and the level of surgical training of the surgical team did not affect anastomotic healing.

Our findings are supported by the results of another study that found inflammation at the resection margins to be quite common with 65.7%. A significant association between positive resection margins and anastomotic leakage could not be demonstrated either in this study [14]. However, this study was not limited to patients with ICR, so a direct comparison with our work is not possible. Our results are indirectly confirmed by the results of two other studies that neither found an association between microscopic inflammation at the resection margins nor a higher rate of anastomotic complications in CD patients [18, 19]. In contrast to our results, the above studies had fewer patient numbers and examined not only the results of ICR in CD patients, but a wider range of resections.

However, another study showed a higher rate of anastomotic related problems including anastomotic leakages and para-anastomotic abscesses in patients with positive resection margins after ICR [7]. In contrast to our study design, this study excluded emergency surgeries. Another study showed higher rates of intra-abdominal complications after bowel resection in CD patients with positive resection margins [20]. The aforementioned study is difficult to compare with ours as it did not focus on anastomotic leakage associated with microscopically inflamed resection margins. Instead, general septic complications and the occurrence of postoperative fistulas were examined. In addition, unlike ours, this study included various abdominal operations. Simultaneous sigmoidectomy, for example, was identified as an independent risk factor for intra-abdominal septic complications. Thus, the comparison is difficult as we focused on ICR as the most common surgery for CD only and excluded multiple bowel resections.

We observed an anastomotic leakage rate of 13.1% in our patient cohort. In the literature, a wide range of values is given for the insufficiency rate according to ICR. There have been reports of 4 to 6% anastomotic leakages after ICR, particularly in studies that excluded emergencies and extended stenosis [8, 21]. Other studies reported a substantially higher leakage rate of 17–19% [6, 22]. These studies often included the analysis of more complex or extended resections and also included emergency procedures. Our hospital is a tertiary referral center for IBD and therefore often treats complex CD patients and emergency cases. This could explain the anastomotic leakage rate that ranges between data from studies with purely elective cases and studies that included complex and emergency procedures.

A limitation of our study is the retrospective study design. However, the underlying question would not be feasible for a randomized design. While a prospective design would be preferable, the groups in our study were homogeneous with regard to underlying diseases and performed operations. In addition, the inclusion of 20% emergency procedures could potentially bias our data. The leakage rate for emergencies was 2 out of 26 (19%) and for non-emergencies 15 out of 104 (14%), so emergencies did not significantly influence the rate of anastomotic leakages in our work. In addition to the limitations mentioned above and since no power analysis was performed, the study potentially was underpowered to detect a difference between the two groups. One of the strengths of the study, however, is the comparatively high number of patients with ICR and CD.

## Conclusion

Microscopic inflammation at the resection margins of ileocecal resections in Crohn´s disease is very common. However, microscopic inflammation this does not appear to play a role in the development of anastomotic leakages. It therefore does not seem to be necessary to perform intraoperative frozen sections and to widen the resection margins to reach areas of the bowel that are not microscopically inflamed. Future larger studies need to confirm these findings.

## Supporting information

S1 Data (XLSX)
